# Dr. Emma W. Moers

**Published:** 1911-07

**Authors:** 


Dr. Emma W. Moers, of the Harvard medical school, died June 1, 1911, at the Massachusetts General Hospital in Boston as a result of streptococcic infection incurred while making pathologic investigation of brain tissues.

